# Efficacy of MP-3 microperimeter biofeedback fixation training for low vision rehabilitation in patients with maculopathy

**DOI:** 10.1186/s12886-022-02419-6

**Published:** 2022-04-28

**Authors:** Tianwei Qian, Xian Xu, Xinyi Liu, Manni Yen, Hao Zhou, Manman Mao, Huiting Cai, Hangqi Shen, Xun Xu, Yuanyuan Gong, Suqin Yu

**Affiliations:** 1 Department of Ophthalmology, Shanghai General Hospital, Shanghai Jiao Tong University, 100 Haining Road, Hongkou District, Shanghai, 200080 China; 2 National Clinical Research Center for Eye Diseases, Shanghai, 200080 China; 3 Shanghai Key Laboratory of Ocular Fundus Diseases, Shanghai, 200080 China; 4 Shanghai Engineering Center for Visual Science and Photomedicine, Shanghai, 200080 China; 5 Shanghai Engineering Center for Precise Diagnosis and Treatment of Eye Disease, Shanghai, China; 6 Shanghai Zhenshi ophthalmology clinic, Shanghai, 200080 China

**Keywords:** Maculopathy, Low vision, MP-3, Microperimeter biofeedback training, Visual rehabilitation

## Abstract

**Background:**

To evaluate the efficacy of MP-3 microperimeter biofeedback fixation training (MBFT) in vision rehabilitation of low-vision patients affected by macular disease with central vision loss.

**Methods:**

Seventeen eyes (7 age-related macular degeneration, 10 myopic maculopathy) of 17 patients were included in this prospective, interventional study. The preferred retinal locus was determined by comprehensive ophthalmoscopic fundus evaluation including fundus photography, autofluorescence, optical coherence tomography, and microperimetry. The rehabilitation consisted of three 10-min sessions per eye to be performed twice per week for 20 consecutive weeks using the MP-3 microperimeter. Best corrected visual acuity (BCVA), reading speed, mean central sensitivity, the percentages of fixation points within specified regions, bivariate contour ellipse area (BCEA) and the 25-item National Eye Institute visual function questionnaire (NEI-VFQ-25) were recorded pre- and post-training.

**Results:**

The final BCVA, reading speed and mean central sensitivity all showed significant improvements after rehabilitation (*P* <  0.0001, *P* = 0.0013, and *P* = 0.0002, respectively). The percentages of fixation points located within 2° and 4° diameter circles both significantly increased after training (*P* = 0.0008 and *P* = 0.0007, respectively). The BCEA encompassing 68.2, 95.4, 99.6% of fixation points were all significantly decreased after training (*P* = 0.0038, *P* = 0.0022, and *P* = 0.0021, respectively). The NEI-VFQ-25 scores were significantly increased at the end of the rehabilitation training (*P* <  0.0001).

**Conclusion:**

Rehabilitation with MP-3 MBFT is a user-friendly therapeutic option for improving visual function, fixation stability, and quality of life in advanced macular disease.

**Trial registration:**

The prospective study was registered with the Chinese Clinical Trial Registry (http://www.chictr.org.cn/). Trial Registration Number: ChiCTR2000029586. Date of registration: 05/02/2020.

## Introduction

The macula is the most sensitive area of the retina, and cone photoreceptors responsible for photopic and color vision are mainly distributed in this area [1]. Macular diseases, such as age-related macular degeneration (AMD) and macular myopic degeneration (MMD), result in irreversible damage and loss of central vision. According to the Taizhou Eye Study, AMD and MMD are the leading causes of low vision using the World Health Organization criteria [2]. In the Western world, AMD is also the leading cause of irreversible loss of central vision in adults over 50 years old [3, 4]. Low vision patients suffer significant loss of abilities such as locomotion, reading, driving, face recognition, and those related to social relationships [5]. No currently available treatment is effective in the reversal of geographic atrophy progression caused by macular lesions [6]. Visual rehabilitation has been widely applied to ocular diseases characterized by visual deterioration and loss of stable central fixation [7] and there is an urgent need to establish and improve the rehabilitation training for macular diseases.

To compensate for loss of central fixation, patients commonly use an eccentric retinal area outside the scotoma, known as the preferred retinal locus (PRL). PRL is defined as an area that contains the center of a target image for over 20% of a fixation interval [8, 9]. Many patients use a PRL in healthy areas of peripheral macula; however, this location is not always ideal and fixation stability is not optimal [10]. Microperimetry may be used to assess PRL and fixation stability [11], classifying the latter as stable, relatively stable, or unstable [12]. Patients with maculopathy regularly demonstrate unstable fixation with associated low vision and the subsequent development of eccentric fixation, with associated brain adaptation strategies [13]. It has been reported that a form of oculomotor exercise known as microperimeter biofeedback training (MBFT), may be used to establish new fixation points and improve fixation stability [10, 14–16]. In patients with macular disease, functional imaging has shown signs of visual cortical reorganization in the areas that correspond topographically to the new fixation [13, 17], and the aim of MBFT training is to strengthen this reorganization to achieve a new stable fixation.

In this study, the MP-3 microperimeter (NIDEK Technologies Srl, Padua, Italy) was used with biofeedback audio signals to aid patients during the MBFT training process by increasing the auditory frequency as the target approaches the desired alignment [18].

The purpose of this study was to evaluate the efficacy of MP-3 MBFT in selecting the best fixation location, improving fixation stability, reading speed, acuity, and quality of life in patients with central vision loss caused by macular diseases.

## Materials and methods

### Patients

This study was conducted in accordance with the Declaration of Helsinki and was approved by the Ethics Committee of Shanghai General Hospital (No. of ethic committee approval: 2018–265). The trial has been registered with Chinese Clinical Trial Registry (ChiCTR2000029586, registration date: 05/02/2020). Written informed consent was obtained from each included patient indicating their agreement to receive microperimeter biofeedback training for vision rehabilitation. All patients underwent a thorough ophthalmic examination, described below. Participants were recruited prospectively and consecutively at the Department of Ophthalmology, Shanghai General Hospital from April 2020 to March 2021. The basic inclusion criteria were as follows: (1) best corrected visual acuity (BCVA) poorer than 20/60 in the better eye; (2) fundus lesions stable and inactive (no ocular treatment received in the preceding 3 months and macular structure stable); (3) willing to complete the whole vision rehabilitation training cycle. The exclusion criteria were as follows: (1) BCVA in the better eye equal to or better than 20/60; (2) central vision loss other than macular atrophic changes; (3) active fundus lesions with bleeding, exudation, and edema; (4) other active eye disease (such as conjunctivitis, uveitis, scleritis, and optic neuritis); (5) opacity of the refractive media such as keratopathy, severe cataract, or severe vitreous opacity; and (6) inability to complete the whole training.

### Ophthalmologic examination and pretraining assessment

All patients underwent complete ophthalmologic evaluation at baseline, including assessment of binocular BCVA using the Early Treatment Diabetic Retinopathy Study (ETDRS) chart, binocular reading speed using the new International Reading Speed Texts (IReST) [19], fundus photography (FP) using the VISUCAM-200 (Carl Zeiss Meditec, Dublin, CA, USA), spectral domain optical coherence tomography (SD-OCT; Spectralis, Heidelberg, Germany), and autofluorescence using the Optos® panoramic 200Tx imaging system (Optos® PLC, Dunfermline, Scotland, UK).

Macular sensitivity and fixation stability were evaluated using the MP-3 microperimeter, which provided a 45° non-mydriatic view of the fundus. Mean central sensitivity and the mean sensitivity at each of 13 central loci within a 2° radius from fixation were recorded pre- and post-training using a 4–2 thresholding strategy with Goldmann III pattern [8]. The MP-3 microperimeter allowed real-time monitoring of fixation 30 times per second using a high-sensitivity and high-speed infrared camera, to achieve automatic correction for eye movement. Retinal light threshold was measured using the Goldmann III with stimulus intensity ranging from 0 to 34 dB and with a stimulus duration of 200 ms.

The 25-item National Eye Institute visual function questionnaire (NEI-VFQ-25) was used to measure the influence of visual disability and symptoms on emotional well-being, social integration, and on task-oriented domains dependent on visual functions [20]. Due to their poor near acuity, all participants were asked the questions verbally by the same investigator (XL).

BCVA, reading speed tests, and the NEI-VFQ-25 test were conducted pre- and post-training to allow evaluation of training effectiveness. These post-training tests would be re-performed half an hour after the last training session on the same day.

### Preparation and training process

The PRL is usually located near the edge of the atrophic retinal area. While this location is sometimes ideal, in most cases it is not optimal. Results from microperimetry and related multimodal images of the target eye indicate that a more optimal area with better visual acuity may be found nearby, and vision rehabilitation training may be used to form a new PRL, known as the target retinal locus (TRL). If there are several regions of the retina with good sensitivity, TRL selection of the dominant eye is based on the following criteria: [6] 1) closest proximity to the fovea and to the PRL; 2) there would be an area with good sensitivity; 3) as horizontally as possible displaced from the PRL in order to facilitate daily visual tasks, especially walking and reading; 4) retinal structure normal and intact according to OCT and autofluorescence.

Vision rehabilitation may be performed with the MP-3 MBFT model to improve fixation stability or relocate the PRL to a more effective position. The MP-3 microperimeter automated program uses a 4–2 thresholding strategy with Goldmann III pattern, and the fixation target size during training is decided by the trainer, an experienced ophthalmologist. In the present study, the standard target was a red cross subtending 1°, but was increased to ≥2° for patients with poorer vision. The training was carried out on the eye with better BCVA and the fellow eye was occluded. An auditory signal present during the training became continuous when fixation was at the optimal location, and intermittent when away from this point. During the training, the trainer instructed the patient on making eye movements to control the auditory signal with the aim of maintaining a continuous signal. Furthermore, patients were encouraged to use this eye movement control in their daily life. The rehabilitation program consisted of three 10-min sessions per eye performed twice per week for 20 consecutive weeks [21].

### Outcome measures

Mean sensitivity at the PRL is an important indicator of retinal function at fixation, and was measured pre- and post-training. Mean sensitivity at PRL was the average of measuring points’ retinal sensitivity within a 2° radius from PRL.

Fixation stability was defined by the percentage of fixation points located within a circle of diameter 2° (FS2°) and 4° (FS4°) centered on all fixation points. Percentage ≥ 75% within the 2° circle indicated stable fixation. Percentage < 75% within 2° and ≥ 75% within 4° indicated relatively unstable fixation and < 75% within 4° indicated unstable fixation [12].

Bivariate contour ellipse area (BCEA) provided a quantitative measure of fixation stability in the area of eccentric PRL. Based on the standard deviations of the horizontal and vertical eye movements during fixation, BCEA was constructed by plotting the position of each fixation on Cartesian axes and calculating the area of an ellipse encompassing 68.2, 95.4, and 99.6% of fixation points [21, 22].

### Statistical analysis

GraphPad Prism (version 6.01, GraphPad software Inc., San Diego, CA, USA) was used for analyses. The Kolmogorov–Smirnov test was used to test for normality of quantitative variables [23]. If normally distributed, the data were expressed as mean and standard deviation (SD) and paired t-tests were applied to compare pre- and post-training data. If not normally distributed, data were expressed as median and interquartile range (IQR), and were compared using the Wilcoxon signed-rank test. All statistical tests were two-sided with *P* < 0.05 considered statistically significant.

## Results

### Patients

In total, 17 eyes from 17 patients (8 male and 9 female) with an average age of 69.41 ± 9.78 years were included in this study. The 17 patients suffered from age-related macular degeneration (8 eyes) or myopic maculopathy (9 eyes). And six patients of them received anti-vascular endothelial growth factor (VEGF) treatment 3 month ago. Baseline characteristics and pre-training assessment of these patients are shown in Table 1.

**Table 1:** Basal characteristics and pretraining assessment of the 17 included patients

Patient	Gender	Age	Diagnosis	Eye	Fixation stability	BCVA (letters)	Mean central sensitivity (dB)	Reading speed (words/min)	FS (%)		BCEA (deg^2^)			NEI-VFQ-25 (scores)
									2°	4°	68.2%	95.4%	99.6%	
1	F	77	MMD	OD	Unstable	36	10.5	98	10.6	29.8	18.4	49.5	94.7	49
2	M	70	AMD	OD	Rel Un	37	16.5	136	40	88.3	6.6	17.8	34	83
3	M	76	AMD	OD	Rel Un	34	10.375	69	43.7	83.9	7.5	20.1	38.4	66
4	M	58	AMD	OS	Unstable	33	13.75	62	11.1	36.8	38.5	103.7	198.5	45
5	M	81	AMD	OD	Rel Un	38	10.25	68	43	86.1	12.8	34.4	65.8	30
6	F	72	MMD	OD	Rel Un	35	7.125	56	33.1	76.1	10.1	27.1	51.9	56
7	F	61	MMD	OS	Unstable	45	5	124	26.4	66.9	13.4	36	69	68
8	F	73	MMD	OD	Unstable	49	9.5	60	14.5	55	16.4	44.2	84.7	38
9	M	59	MMD	OS	Unstable	47	2	98	1.3	23.5	37.8	101.7	194.6	60
10	M	69	MMD	OS	Unstable	50	13.875	107	6.3	23.1	45.7	123.1	235.7	68
11	M	82	AMD	OD	Unstable	37	13.875	68	11.3	50.4	25	67.2	128.6	46
12	F	70	MMD	OS	Unstable	38	5.25	45	66.6	95.7	3.7	95.4	99	60
13	F	74	AMD	OD	Unstable	33	11.375	85	13.3	44.7	20.8	55.9	106.9	33
14	F	63	MMD	OD	Rel Un	37	2.875	75	63.1	86.7	4.7	12.7	24.2	62
15	M	44	AMD	OS	Rel Un	35	16.25	68	38.9	79.9	6.7	18	34.5	65
16	F	72	MMD	OD	Rel Un	45	7.75	70	8.6	39	18.5	49.9	95.5	63
17	F	79	MMD	OS	Rel Un	39	14.5	82	56.9	85.9	5.5	14.8	28.3	78

*AMD* Age-related macular degeneration, *MMD* Myopic maculopathy degeneration, *Rel Un* Relatively unstable

During the training process, we changed the PRL into TRL in 6 eyes and we use the same PRL in another 11 eyes. All 17 participants completed the rehabilitation training program at the end of which fixation had changed in all cases from poor central fixation to predominantly central fixation, and 7 of the 17 eyes showed an improvement in fixation stability from unstable to relatively unstable or stable.

Table 2 shows pre-post training comparisons of main outcomes. BCVA (*P* < 0.0001), mean central sensitivity (*P* = 0.0002) and reading speed (*P* = 0.0013) all improved significantly after training. Fixation stability was significantly increased at 2° and 4° (*P* = 0.0002 and *P* = 0.0007 respectively) and BCEAs encompassing 68.2, 95.4, 99.6% of fixation points were significantly decreased after training (*P* = 0.0038, *P* = 0.0022, and *P* = 0.0021, respectively).

**Table 2:** Outcome measures pre- and post-rehabilitation training

Parameters	Pre-training	Post-training	***P*** value
BCVA (letters)	39.29 ± 5.63	42.24 ± 5.39	***< 0.0001***
Mean central sensitivity (dB)	10.04 ± 4.50	14.35 ± 4.83	***0.0002***
Reading speed (words/min)	80.65 ± 24.59	88.71 ± 25.9	***0.0013***
FS 2° (%)	26.4 (31.9)	52.5 (20.9)	***0.0002***^***#***^
FS 4° (%)	66.9 (46.9)	88.9 (9.0)	***0.0007***^***#***^
BCEA 68.2% (deg^2^)	17.18 ± 12.8	6.80 ± 4.90	***0.0038***
BCEA 95.4% (deg^2^)	51.26 ± 35.22	18.36 ± 13.14	***0.0022***
BCEA 99.6% (deg^2^)	93.19 ± 63.84	35.13 ± 25.14	***0.0021***
NEI-VFQ-25 (scores)	57.06 ± 14.96	65.59 ± 16.88	***< 0.0001***

Data are expressed as mean ± standard deviation or median (interquartile range) for normal and non-normal distributions, respectively. *BCVA* Best corrected visual acuity, *FS* fixation stability, *BCEA* Bivariate contour ellipse area, *NEI-VFQ-25* National Eye Institute Visual Functioning Questionnaire 25-item questionnaire

# Wilcoxon signed-rank test was used to analyze the pre-post training differences

**Fig. 1:**
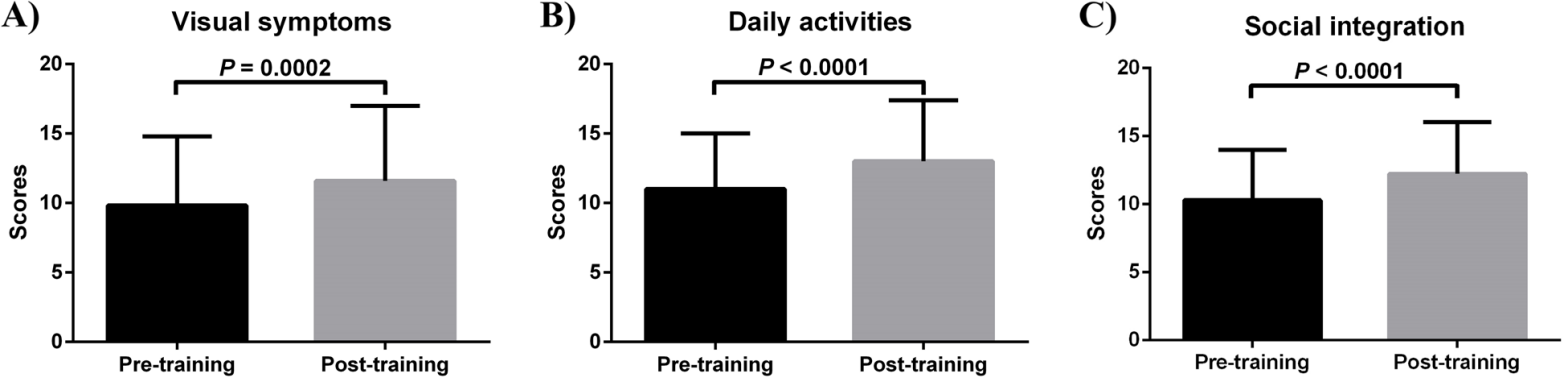
Pre-post training changes in visual symptoms (**A**), daily activities (**B**) and social integration scores (**C**)

Mean NEI-VFQ-25 scores were also increased (*P* < 0.0001) with significant improvements in visual symptoms, daily activities, and social integration indicated by the relevant items in the questionnaire (Fig. 1).

**Fig. 2:**
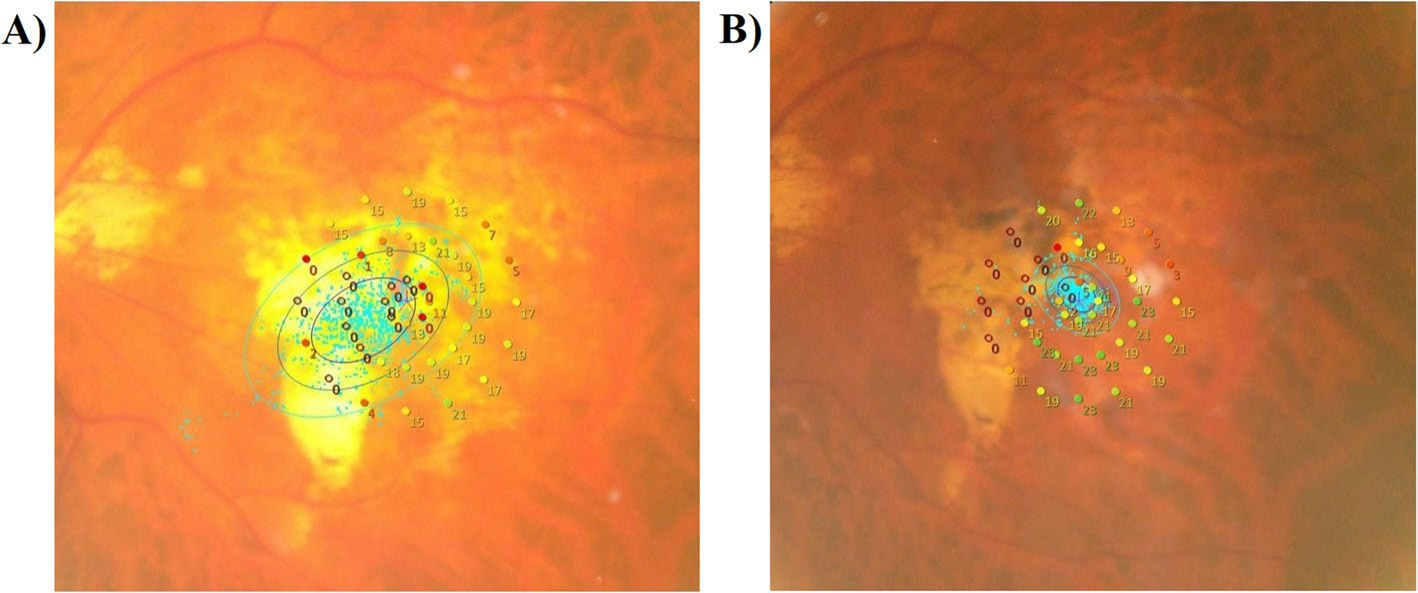
The microperimetry results of a 61-year-old female with macular myopic degeneration of the left eye. **A** Pre-training; **B** Post-training. The fixation point position successfully moved to the selected area and three BCEAs also decreased after training

Figure 2 shows the microperimetry results of a 61-year-old female with macular myopic degeneration of the left eye before (A) and after (B) training. After training, the fixation point position had moved successfully from the atrophic area of the retina to the selected area and the fixation stability changed from unstable to stable. BCVA and reading speed were also improved after training.

## Discussion

Various macular diseases may cause structural damage to the fovea with loss of central vision, including low visual acuity and unstable fixation [6]. To date, no medical intervention can effectively reverse the loss of macular function. Therefore, interventions that may help to modify the visual system and improve visual acuity and quality of life deserve attention [24]. Microperimetry training is one such approach which works by selecting, training, and stabilizing the PRL [7, 24]. For this reason, we evaluated the efficacy of MP-3 MBFT as a means of improving fixation stability and as a rehabilitation program in patients with low vision secondary to maculopathy. Our results, similar to those of some previous studies [25–27], showed that rehabilitation with MP-3 MBFT is a useful means by which to improve BCVA, reading speed, fixation stability, and quality of life, bringing hope for vision rehabilitation of low vision patients.

This approach trains patients to fixate the target with a new PRL. During the training process, eye movement is monitored by the device and the patients are reminded by audio feedback whether their fixation is near to the pre-selected specific retinal region chosen by experienced ophthalmologists. In addition to time availability, this training requires that the patients, especially the elderly, should have good comprehension skills and understanding of the training process [6]. The training helps patients to increase their awareness of their fixation and its stability through audio feedback.

Microperimeter biofeedback training has been used in many areas of rehabilitation of the visually impaired, such as amblyopia, strabismus, and nystagmus, for at least twenty years [28, 29]. Retinal sensitivity improvement after training with microperimetry has been reported in a sample of five patients with different macular diseases [30]. Microperimetry is also a useful tool to evidence decreased retinal sensitivity and fixation quality in AMD progression [26]. Based on visual evoked potential (VEP) real-time examination, biofeedback rehabilitation has proven useful in the improvement of visual acuity, reading performance, and quality of life in AMD patients [31]. For patients with Stargardt disease, MBFT increases quality of vision, leading to stabilized fixation and a consequent improvement in patients’ visual function and reading speed [24, 27, 32]. Daibert-Nido et al also reported that biofeedback training may offer patients a unique and efficient modality to improve distance vision [33].

Apart from the NIDEK MP-3 microperimeter biofeedback training system, the Visual Pathfinder (LACE Inc., Rome, Italy) rehabilitation system has also demonstrated improvements in BCVA, amplitude of the main peak of the pattern reversal VEP, fixation behavior and retinal sensitivity in patients with high myopia and retinitis pigmentosa [34].

However, the mechanism underpinning modification of the PRL and improvements in visual perception remains unclear. At present, a reasonable explanation of this improvement is that training re-references the oculomotor system to a location with improved visual sensitivity [24]. When there is local pathological damage to the retina, while the affected area cannot be stimulated, cortical neurons normally driven by stimuli in this area remain active and respond selectively to stimulation from other parts of the retina [35, 36]. MBFT, with its auditory feedback, may help to sustain target stimulation of the retina, thus strengthening the patient’s cortical plasticity and facilitating neural signals within the retina and between the retina and brain [37, 38], a phenomenon termed “neuro-remapping” [39, 40]. Due to the long-term existence of a central scotoma in irreversible macular foveal injury, adaptation will require the brain to adopt strategies to form an alternative fixation site with improved visual function. The stabilization of PRL also contributes to the reactivation of brain areas involved in central vision [9, 32]. This process is long and unpredictable and MP-3 biofeedback training aims to shorten its duration and increase its predictability.

In our study, BCVA improved after training in all patients. Better visual acuity at the TRL means less eye movement and this fixation stability reinforces improvement in acuity.

One limitation of this study is the small sample size. During the COVID-19 epidemic, the recruitment of patients was challenging, and the absence of stratification by disease is a limitation. The lack of a control group is another limitation of the study. However, our results can provide a preliminary basis for large-scale controlled trials in the future. The third limitation is the follow-up time which was relatively short and did not allow long-term assessment of fixation stability. Our future research will investigate the long-term effectiveness of the approach used here, particularly with regard to visual function changes in progression of different diseases.

## Conclusion

Biofeedback training using the MP-3 microperimeter is effective in improving visual acuity, reading speed, fixation stability, and quality of life in patients with maculopathy. After selecting optimal fixation, the training facilitates adaptation of the PRL, maximizing patients’ remaining vision and their fixation stability. Although the exact mechanism of this improvement remains unclear and needs further exploration, MP-3 MBFT is an effective, repeatable, and noninvasive method which brings hope to patients who suffer from atrophic maculopathy and have had no effective treatment option to date.

## Data Availability

The datasets generated and/or analyzed during the current study are not publicly available due the protection of the rights and interests of visual disablement patients by the Ethics Committee of Shanghai General Hospital but are available from the corresponding author on reasonable request.

## Abbreviations

MBFT: Microperimeter biofeedback fixation training
BCVA: Best corrected visual acuity
BCEA: Bivariate contour ellipse area
NEI-VFQ-25: The 25-item National Eye Institute visual function questionnaire
AMD: Age-related macular degeneration
MMD: Macular myopic degeneration
PRL: Preferred retinal locus
ETDRS: Early Treatment Diabetic Retinopathy Study
IReST: International Reading Speed Texts
FP: Fundus photography
OCT: Optical coherence tomography
FS: Fixation stability
SD: Standard deviation
IQR: Interquartile range
